# The complete chloroplast genome of tropical and sub-tropical fruit tree *Lucuma nervosa* (Sapotaceae)

**DOI:** 10.1080/23802359.2018.1457995

**Published:** 2018-04-03

**Authors:** Ying-Feng Niu, Shu-Bang Ni, Zi-Yan Liu, Cheng Zheng, Chang-Li Mao, Chao Shi, Jin Liu

**Affiliations:** a Yunnan Institute of Tropical Crops, Jinghong, PR China;; b Kunming Institute of Botany, Chinese Academy of Sciences, Kunming, PR China

**Keywords:** Chloroplast genome, *Lucuma nervosa*, Sapotaceae

## Abstract

The *Lucuma nervosa*, native to Western Ghats of India, Malaysia and south-eastern Asia, is a tree member of the mulberry family (Sapotaceae). Chloroplast genome sequences play an significant role in the development of molecular markers in plant phylogenetic and population genetic studies. In this study, we report the complete chloroplast genome sequence of *L. nervosa* for the first time. The chloroplast genome is 157,920 bp long and includes 113 genes. Its LSC, SSC, and IR regions are 88,123, 18,861, and 25,468 bp long, respectively. Phylogenetic tree analysis exhibited that *L. nervosa* was clustered with other Sapotaceae species with high bootstrap values.

The *Lucuma nervosa* is a perennial evergreen fruit tree of the family Sapotaceae. It is native to the tropics of Cuba and South America, and are cultivated sporadically in Guangdong, Guangxi, southern Yunnan and Hainan of China (Liu et al. 2013). Its fruit can be eaten fresh or processed into fruit paste and cream with edible rate above 70%, which was rich in nutrition, including soluble solids 14.48–17.53%, sugar 29.1–30.5%, starch 5.6–8.1%, crude fat 1–1.4%, every 100 g flesh contains vitamin C 29.38–57.55 MG, acid content of 0.079–0.165% (Deng et al. 2013). Furthermore, the trees are beautiful and can be used for ornamental cultivation (Deng et al. 2013).

There was only one complete chloroplast genome reported in the family Sapotaceae (Jo et al. 2016). In this study, we report the complete chloroplast genome of *L. nervosa*, the second complete plastome sequence from the family Sapotaceae. DNA material was isolated from mature leaves of a *L. nervosa* plant cultivated in the plant garden of Yunnan Institute of Tropical Crops (YITC), Jinghong, China by using DNeasy Plant Mini Kit (QIAGEN, Hilden, Germany). A specimen of this tree was conserved in YITC. About 10 μg isolated DNA was sent to BGI, Shenzhen for library construction and genome sequencing on the Illumina Hiseq 2000 Platform. After genome sequencing, a total of 3.2 Gbp reads in fastq format were obtained and subjected to chloroplast genome assembly. The complete chloroplast genome was annotated with Dual Organelle GenoMe Annotator (DOGMA; Wyman et al. 2004) and submitted to the GenBank under the accession number of MH018545.

Our assembly of the *L. nervosa* resulted in a final sequence of 157,920 bp in length with no gap. The overall A–T content of the chloroplast genome was 61.2%. This chloroplast genome included a typical quadripartite structure with the large single copy (LSC), small single copy (SSC), and inverted repeat (IR) regions of 88,123, 18,861, and 25,468 bp long, respectively. Genome annotation showed 113 full length genes including 79 protein-coding genes, 30 tRNA genes, and four rRNA genes. The genome organization, gene content, and gene relative positions were almost identical to the previously reported Sapotaceae chloroplast genomes (Jo et al. 2016). To validate the phylogenetic relationships of *L. nervosa* in the Ericales, we constructed a maximum likelihood tree using 14 super-Ericales taxa. Phylogenetic analysis was performed on a data set that included 79 protein-coding genes and four rRNA genes from the 27 selected taxa using RAxML v. 7.7.1 (Stamatakis et al. 2008). The 83 gene sequences (82,565 bp in length) were aligned with the MAFFT (Katoh and Standley 2013). The resulting tree shows that *L. nervosa* forms a clade with the species of Ebenaceae and Primulaceae with a 100% bootstrap value (Figure 1). The Sapotaceae represented by *L. nervosa* form a sister group to the Primulaceae–Ebenaceae clade within the Ericales. The close relationship of Sapotaceae to Lecythidaceae was suggested by a previous study (Anderberg et al. 2002).

**Figure 1.**
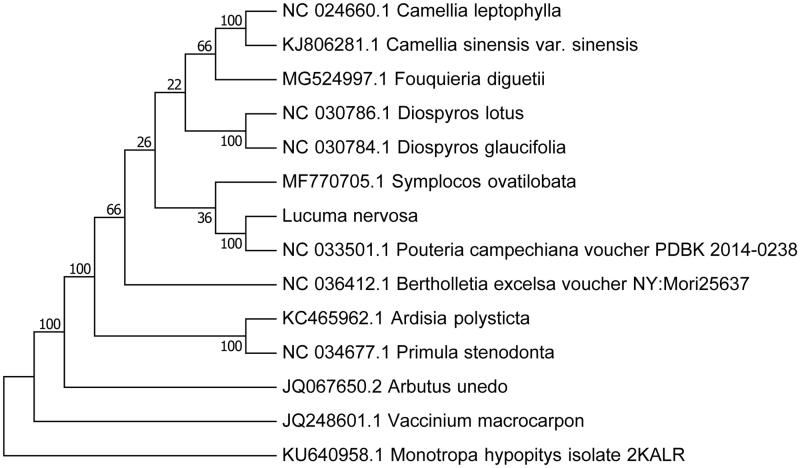
Maximum-likelihood (ML) phylogenetic tree of *L. nervosa* in the Ericales. Number above each node indicates the ML bootstrap support values.
